# Treatment Strategies Based on Histological Targets against Invasive and Resistant Glioblastoma

**DOI:** 10.1155/2019/2964783

**Published:** 2019-06-20

**Authors:** Akira Hara, Tomohiro Kanayama, Kei Noguchi, Ayumi Niwa, Masafumi Miyai, Masaya Kawaguchi, Kazuhisa Ishida, Yuichiro Hatano, Masayuki Niwa, Hiroyuki Tomita

**Affiliations:** ^1^Department of Tumor Pathology, Gifu University Graduate School of Medicine, 1-1 Yanagido, Gifu City, Gifu 501-1194, Japan; ^2^Department of Neurosurgery, Gifu University Graduate School of Medicine, 1-1 Yanagido, Gifu City, Gifu 501-1194, Japan; ^3^Department of Radiology, Gifu University Graduate School of Medicine, 1-1 Yanagido, Gifu City, Gifu 501-1194, Japan; ^4^Department of Oral and Maxillofacial Science, Gifu University Graduate School of Medicine, 1-1 Yanagido, Gifu City, Gifu 501-1194, Japan; ^5^Medical Science Division, United Graduate School of Drug Discovery and Medical Information Sciences, Gifu University, 1-1 Yanagido, Gifu City, Gifu 501-1194, Japan

## Abstract

Glioblastoma (GBM) is the most common and the most malignant primary brain tumor and is characterized by rapid proliferation, invasion into surrounding normal brain tissues, and consequent aberrant vascularization. In these characteristics of GBM, invasive properties are responsible for its recurrence after various therapies. The histomorphological patterns of glioma cell invasion have often been referred to as the “secondary structures of Scherer.” The “secondary structures of Scherer” can be classified mainly into four histological types as (i) perineuronal satellitosis, (ii) perivascular satellitosis, (iii) subpial spread, and (iv) invasion along the white matter tracts. In order to develop therapeutic interventions to mitigate glioma cell migration, it is important to understand the biological mechanism underlying the formation of these secondary structures. The main focus of this review is to examine new molecular pathways based on the histopathological evidence of GBM invasion as major prognostic factors for the high recurrence rate for GBMs. The histopathology-based pharmacological and biological targets for treatment strategies may improve the management of invasive and resistant GBMs.

## 1. Introduction

**Figure 1:**
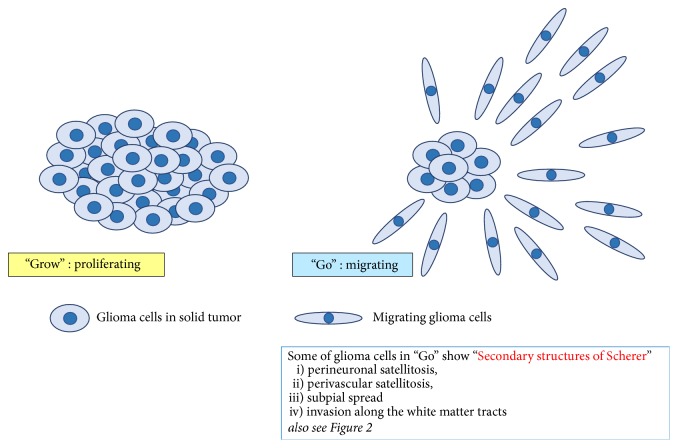
Illustration of “Go or Grow” theory in malignant gliomas. Malignant gliomas often consist of two subpopulations of cells, which mutually interact and mutually change, that are characterized by uncontrolled-proliferation and by abnormal migration. One subpopulation of cells is rapidly proliferating and forming a stationary tumor mass, while the other subpopulation is actively migrating and moves into surrounding brain without cell division. Some of glioma cells in “Go” stage show characteristic morphological patterns of tumor cell migration, referred to as “secondary structures of Scherer.” These “secondary structures of Scherer,” which are also shown in Figure 2, have been classified into histological patterns: (i) perineuronal satellitosis, (ii) perivascular satellitosis, (iii) subpial spread, and (iv) invasion along the white matter tracts.

**Figure 2:**
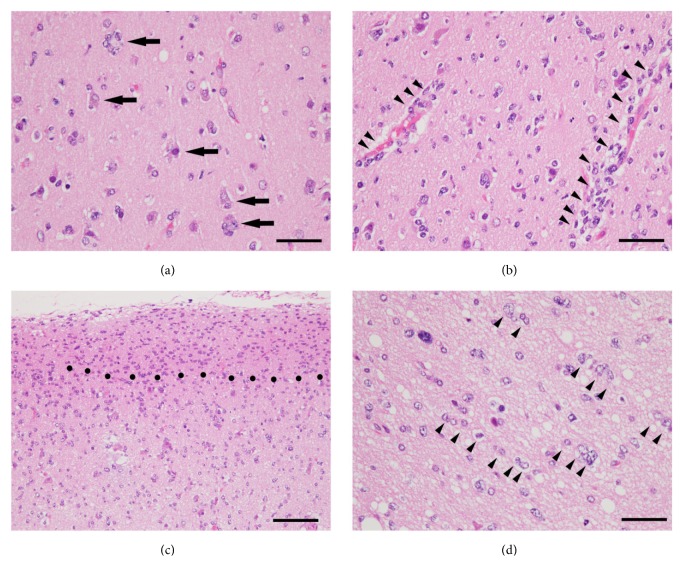
Specific histomorphological patterns of diffuse invasion, so-called “secondary structures of Scherer” in glioblastoma. As a rule, glioma cells migrate along existing brain structures such as brain parenchyma, blood vessels, white matter tracts, and subpial spaces. The secondary structures of Scherer are referred to four criteria as (a) perineuronal satellitosis (indicated by arrows), (b) perivascular satellitosis (indicated by arrow heads), (c) subpial spread (region above black dots), and (d) invasion along the white matter tracts (indicated by arrow heads). Hematoxylin and eosin staining. Scale bars in (a), (b), and (d) are 50 *μ*m; scale bar in (c), 100 *μ*m.

Glioblastoma (GBM) is the most invasive, infiltrative, and lethal brain tumor with high proliferative potential [1]. Malignant gliomas, also called as high-grade gliomas and including GBM (WHO grade IV gliomas) and anaplastic gliomas (WHO grade III gliomas), are currently incurable despite aggressive surgery and are resistant to conventional therapies. Patient outcome following standard therapies including radiation and chemotherapy for GBM remains poor, with a median overall survival of only 12–14 months [2]. The highly invasive tumor cells predominantly migrate out of the tumor mass into the surrounding normal central nervous system. And they escape surgical resection and resist conventional treatments such as radiation and temozolomide, both of which are the first line of treatment for GBM patients following surgery. The surviving glioma cells after conventional therapies that target proliferating cells are principally responsible for tumor recurrence. Therefore, the effective treatment strategies which improve the management of invasive and resistant GBM cells are urgently needed to manage this malignancy. Histopathologically, infiltrated GBM cells show some specific morphological patterns, characterized as diffuse invasion. In general, glioma cells migrate along existing brain structures such as the brain parenchyma, blood vessels, white matter tracts, and subpial spaces. These characteristic morphological patterns of tumor cell migration from the growing tumor mass into the adjacent brain tissues have been described first by Hans Joachim Scherer in 1938 [3] and referred to as “secondary structures of Scherer.” These “secondary structures of Scherer” have been classified into histological patterns: (i) perineuronal satellitosis, (ii) perivascular satellitosis, (iii) subpial spread, and (iv) invasion along the white matter tracts (Figures 1 and 2). Careful observations of these histomorphological features have revealed the important contributions of the microenvironment that influence glioma cell migration. It is possible that invasive glioma cells showing “secondary structures of Scherer” mimic key intracellular processes of both proliferation and migration that occur in neural stem cells or glial progenitor cells within the developing central nervous system [4].

## 2. Similarities between Tumor Invasiveness of GBMs and Migrating Characteristics of Stem Cells

### 2.1. Stem Cells in Normal Brain

During neural development, neurons and glia are generated from developmental stage-specific and rapidly-dividing progenitor cells, and then quiescent, multipotent stem cells remain stable throughout adulthood [5]. During the formation of embryonic cerebral cortex, newly formed neuronal and glial progenitor cells migrate from the originating periventricular zone toward their specified physiological locations throughout the central nervous system [6–8]. Even in the adult mammalian brain, the subventricular zone (SVZ) contains neural stem cells (NSCs) or progenitor cells, which continue to produce neurons or glia for the maintenance of the central nervous system [7]. The SVZ of rodents, monkeys, and humans is a source of NSCs during adult neurogenesis. Some pathophysiological conditions, such as epilepsy [9], stimulate neurogenesis in the rostral forebrain SVZ of adult rats. Following ischemic insult, neural progenitors in caudal SVZ migrate to the hippocampus and contribute to the pyramidal cell regeneration in hippocampal CA1 [9]. Furthermore, some recent studies [10] suggest that the SVZ may also provide a source of brain tumor stem cells, which are morphologically and physiologically similar to NSCs producing neurons, astrocytes, and oligodendrocytes.

### 2.2. Relationship between SVZ and Brain Tumors

Unlike in normal brain development, tumor progenitor and quiescent tumor stem cell populations have yet to be understood in brain tumors [5]. A similarity between production of NSC in SVZ and the generation of malignant gliomas has been suggested [10]. While the underlying mechanisms remain unclear, it has been suggested that increased tumor invasiveness, early recurrence, and mortality are worse in those patients whose malignant gliomas infiltrate or contact with the SVZ [11, 12]. It is hypothesized the SVZ has a unique role of contribution to GBM tumorigenesis in adult brain. Thus, these findings strongly suggest that the invasiveness of malignant glioma is derived from the migratory nature of NSCs in the adult human brain and that the morphological structures formed by invasive GBM, the “secondary structures of Scherer,” are based on the functional similarities of invasive GBM to developing NSCs.

### 2.3. Glioma-Derived Cancer Stem Cells

Evidence suggests that the invasiveness of malignant gliomas, which mimic the migration attributes of NSCs, could be therapeutically controlled by modifying the intracellular systems or molecular pathways. Moreover, glioma-derived cancer stem cells (CSCs), which are regarded to be a counterpart of primary NSCs in normal brain, have subsequently been shown to be resistant to chemotherapy [13] and radiotherapy [14]. Glioma CSCs are key players in tumor initiation, therapeutic resistance, and tumor recurrence [4, 15–22] and are also related to glioblastoma heterogeneity. GBM is genotypically and phenotypically heterogeneous, which is a major factor in its poor response to various therapies. It is reported that even single cell-derived subclones from a patient can produce phenotypically heterogeneous self-renewing progenies in both* in vitro* and* in vivo* settings [23]. Several therapeutic strategies targeting glioma CSCs have been proposed to effectively control the disease progression [24–30].

## 3. Gliomatosis Cerebri and GBM

### 3.1. Gliomatosis Cerebri

Gliomatosis cerebri (GC) is an extremely rare neoplasm which shows diffuse infiltration of glioma cells within central nervous system including brain as well as spinal cord. Since clinical manifestations are various and focal neurological signs are usually recognized late in the course of the disease, the early recognition of this disease is difficult [31, 32], and no standard of care is usually available for the treatment of GC patients. Historically, histopathologic diagnosis of GC has been determined using standard hematoxylin and eosin staining and immunohistochemistry, with GC being defined as a distinct pathologic entity in “World Health Organization (WHO) Classification of Tumors of the Central Nervous System” up to the 3rd edition. However, the scientific consensus now is that there is no common pathologic and radiographic consensus for its diagnosis, and in 2016, GC was eliminated from current WHO classification [33]. The reason of the elimination was based on overlap of discrete molecular alterations with other malignant gliomas and the absence of specific molecular markers [34, 35]. Many histomorphological features similar to infiltrative gliomas support the contention that GC is one variety of diffuse glioma including GBM. Therefore, GC is currently considered to be an extremely infiltrative subtype of diffusely growing malignant glioma, instead of a distinct histologic or molecular subtype of glioma.

### 3.2. Histological Findings of Gliomatosis Cerebri

**Figure 3:**
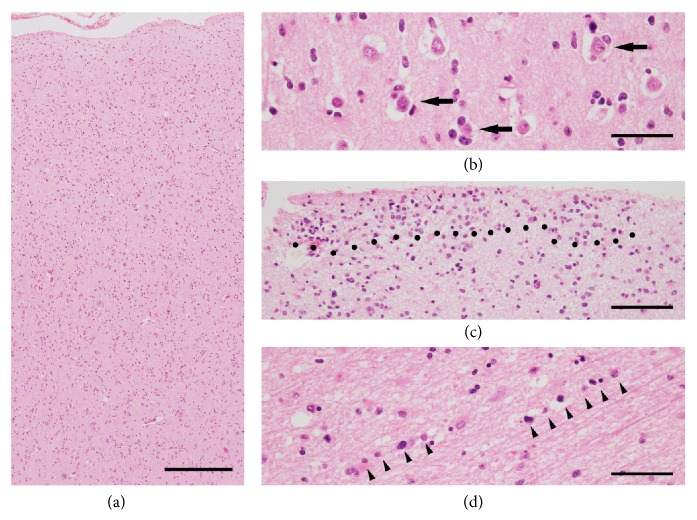
Representative images showing histomorphological structures of GC. Many histological features are similar to invasive patterns of GBM (secondary structures of Scherer in Figure 2). (a) Diffuse parenchymal infiltration of GC cells without the formation of a circumscribed tumor mass. The secondary structures of Scherer are seen also in GC as follows: (b) perineuronal satellitosis (indicated by arrows), (c) subpial spread (region above black dots), and (d) invasion along the white matter tracts (indicated by arrow heads). Hematoxylin and eosin staining. Scale bar in (a), 375 *μ*m; scale bars in (b) and (d), 50 *μ*m; scale bar in (c), 100 *μ*m.

GC has specific histological features, namely, tumor cells (i) that are elongated with diffuse and irregular parenchymal infiltration (without formation of a circumscribed tumor mass), (ii) with perivascular or perineuronal satellitosis, (iii) with subpial spread, and (iv) that infiltrate along myelinated tracts with the preserved neuronal axons (Figure 3). Neoplastic cells with elongated and fibrous cell processes were recognized easily with glial fibrillary acidic protein (GFAP) immunohistochemistry [31]. Since the anaplastic single cells infiltrate along myelinated axons and the basement membranes of blood vessels with distinct anatomic structures, typical features of GBM (e.g., neovascularization, necrosis, and mitotic activity) are usually absent in the lesions. The overall delineation of the histological findings for GC is similar to “secondary structures of Scherer” in the lesion of cell migration in malignant glioma.

### 3.3. Gliomatosis Cerebri and “Go or Grow”

Usually, the neoplastic cells in GC do not have much proliferative activity, similar to that of low-grade gliomas [31, 36]. The fact that the neoplastic cells of GC infiltrate with low proliferative activity into brain tissues is very consistent with the following hypothesis, “Go or Grow” dichotomy theory.

## 4. “Go or Grow” Theory in Malignant Gliomas

### 4.1. Two Subpopulations

**Figure 4:**
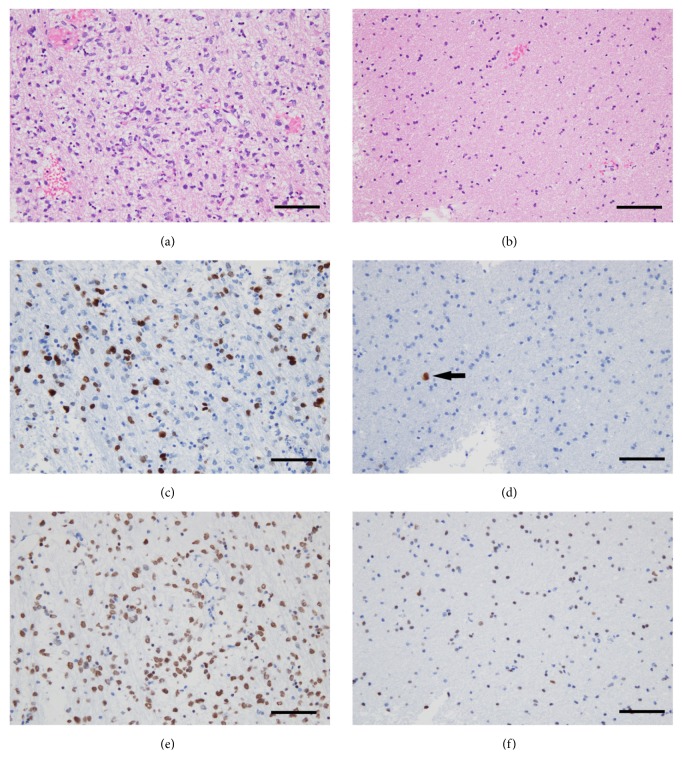
Representative histomorphological features of “Go or Grow” in GBM. The two subpopulations consist of uncontrolled-proliferating and abnormally migrating cells which interact mutually, which is so-called ‘‘Go or Grow” in gliomas. One subpopulation, rapidly proliferating cells, forms tumor mass being stationary (a, c, e). The other subpopulation, actively migrating cells, moves into surrounding brain without cell division (b, d, f). (a, b) Hematoxylin and eosin staining. (c, d) Immunohistochemistry for Ki-67 antigen, a marker of proliferating cells. The Ki-67 positive cells showing dark brown cell nuclei are detected as proliferating cells. (e, f) Immunohistochemistry for oligodendrocyte transcription factor (OLIG2), which is expressed universally in GBM cell nuclei. Vascular cells in GBM (observed in (e)) and normal glia cells (observed in (f)) are negative for OLIG2. Note that only one Ki-67 positive cell is detected in migrating GBM cells (arrow in (d)), whereas many OLIG2 positive GBM cells are seen in the same area (f). Also note that the sizes of cell nuclei recognized in (f) are smaller than that in (e). This means that migrating GBM cells into surrounding brain have smaller nuclei because they are actively moving. Scale bars, 100 *μ*m.

Malignant gliomas often consist of two subpopulations of cells, which mutually interact and mutually change, that are characterized by uncontrolled-proliferation and by abnormal migration. This has been termed the “Go or Grow” theory of gliomas (Figure 1). One subpopulation of cells is rapidly proliferating and forming a stationary tumor mass, while the other subpopulation is actively migrating and moves into surrounding brain without cell division (Figure 4). It has been hypothesized that cell proliferation and cell migration in gliomas are distinct and mutually exclusive, with a trade-off between them [37–43]. Tumor microenvironment and the metabolic stress (hypoxia; glucose deprivation) may regulate the switching between Go and Grow behaviors in GBM. It is possible that the surviving and invasive glioma cells after conventional therapies, which show “Go behavior,” may later switch to a proliferative “Grow phenotype” at satellite lesions, forming rapid tumor mass.

### 4.2. Mechanism

This highly complex phenomenon involving molecular and cellular processes has been extensively studied. The mechanisms mediating uncontrolled-proliferation and abnormal migration of glioma cells have been investigated using glioma cell cultures. For example, the population of nonmotile glioma cells in standard cell culture conditions exhibits decreased intercellular space and high proliferating activity, indicating that the population is undergoing cell growth and division. In contrast, cells cultured on laminin exhibit activate migration, enlargement of the intercellular space, and spreading away from the proliferating growth site [44]. Glioma cells growing at the tumor core have a high proliferative activity, whereas migrating/invading cells around the tumor demonstrate a low proliferative activity, with NF-kB activated in migration-stimulated GBM cells and c-Myc activated in migration-restricted GBM cells [40].

### 4.3. Spheroid Analysis

Glioma invasion* in vivo* and* in vitro* differs in several ways. Monolayer cell culture is typically employed for* in vitro* experiments for many kinds of tumor cells, but employing tumor cell spheroids in a three-dimensional culture is better model to mimic* in vivo *conditions. A spatiotemporal spheroid analysis of U87 glioma cells showing high invasiveness, using computational and experimental approaches, implicates intrinsic cellular mechanisms of glioma invasion: local cell density, radial oriented cell motion away from the spheroid, and intercellular repulsion dynamics are involved in glioma invasion [45].

### 4.4. Mathematical Model

Recently, a mathematical model based on the proliferation/migration dichotomy of glioma cells has been applied to investigate why modulatory interventions against glioma vascularization have not been successful at controlling glioma invasion [46]. The study found that cell proliferation/migration ratio was a critical determinant of glioma responses to vasomodulatory interventions against glioma vascularization [46].

### 4.5. Therapeutic Approach

The “Go or Grow” potential of gliomas is related to metabolic stress which mediates some neuropeptide-processing enzymes. Reduced expression of carboxypeptidase E (CPE), a neuropeptide-processing enzyme that is induced by environmental stressors such as hypoxia and glucose deprivation, contributes to GBM cell migration and invasion [47]. The study indicates that loss or reduction of CPE expression correlated with poor prognosis of GBM patients. The control of metabolic stress based on the “Go or Grow” hypothesis may be a potential target for future antiglioma therapeutic approach mediating glioma biology.

The mitotic kinesin, KIF11, is a driver of glioblastoma invasion, proliferation, and self-renewal [48]. Inhibition of KIF11 with a highly specific small-molecule inhibitor regulates GBM cell growth and motility, associated with intratumoral heterogeneity, suggesting that KIF11 is a therapeutic target for glioblastoma treatment.

Further analysis of the cellular, molecular, and genetic processes underlying the “Go or Grow” dichotomy is warranted to elucidate novel therapeutic approaches for glioma invasion.

## 5. Overview of Animal Model for Glioma

The invasiveness of human malignant brain tumors has been reproduced in several animal models in order to investigate detailed histopathological processes.

### 5.1. N-Ethyl-N-Nitrosourea-Induced Rat Glioma Model

One useful animal model is N-ethyl-N-nitrosourea- (ENU-) induced rat gliomas. A high incidence of CNS tumors including GBMs has been consistently induced in the offspring of rats treated with a single dose of transplacental ENU [49], and many aspects of this model have been studied [50–52]. A size-oriented classification for ENU-induced rat glial tumors has been established: early neoplastic proliferation (ENP), microtumors, and macrotumors [49, 52, 53]. ENP represents a focus of glial population less than 300 *μ*m in diameter. Due to their small size, it is difficult to identify ENPs histomorphologically by conventional hematoxylin and eosin staining; therefore they are immunohistochemically using galectin-3 antibody [52]. Microtumors, exhibiting destructive histopathological features, are distinguished from macrotumors by their size, being between 300 and 500 *μ*m in diameter. The macrotumors are considered to be an advanced stage of the neoplastic growth process, and when induced by ENU, they are used as endogenously produced gliomas for analyses of invasiveness [54].

### 5.2. Genetically Engineered Animal Models

Genetically engineered animals, one of the powerful tools for studying the biology of neoplasms and oncogene identification, have also been employed as developing mouse glioma models. Genetically engineered mice have been successfully used to investigate tumorigenesis and its progression within an intact living organ as animal models for human neoplasms. Weissenberger et al. [55] generated a transgenic mouse model showing the overexpression of v-src oncogene under the control of GFAP regulatory promotor. The transgenic mice produced low-grade astrocytomas in early phase and high-grade astrocytomas in later phase of glioma tumorigenesis. The morphological characteristics such as pseudopalisading cells surrounding necrotic areas were induced in GBM with high mitotic activity. Genetically engineered histone H3 K27M mutations in neonatal mice cooperate with activating platelet-derived growth factor receptor *α* (PDGFR*α*) mutant and Trp53 loss that may induce self-renewal of neural stem cell and develop diffuse intrinsic pontine glioma (DIPG) recapitulating human DIPG [56]. The histone H3 K27M mutation appears fundamental and important event in diffuse infiltration of glioma cells in DIPG.

### 5.3. Human Xenograft Glioma Models

**Figure 5:**
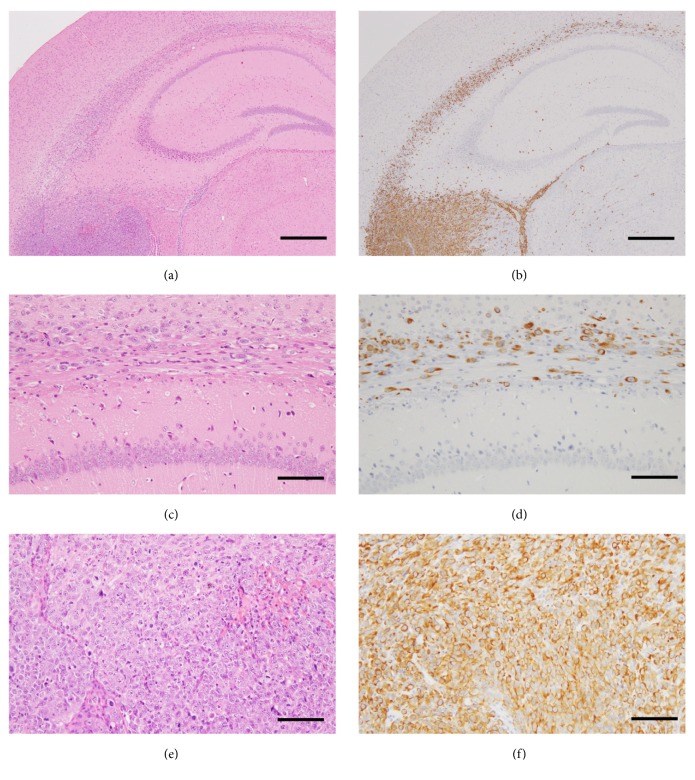
The human GBM orthotopic xenograft exhibiting invasive and extensive infiltration in NOD-scid mice. The nestin-expressing NSC-like GBM cells are highly invasive, showing diffuse infiltration into the brain including the corpus callosum, hippocampus, and the subependymal regions. (a, b) Photomicrographs of low-power field of human GBM orthotopic xenograft in NOD-scid mouse brain. (c, d) Photomicrographs of high-power field of corpus callosum infiltrated by human GBM. (e, f) Photomicrographs of high-power field of mass lesion of human GBM. (a, c, e) Hematoxylin and eosin staining. (b, d, f) Immunohistochemistry for nestin. Scale bars in (a) and (b), 500 *μ*m; scale bars in (c), (d), (e), and (f), 100 *μ*m.

Animal models of human GBMs have been established, including subcutaneous or orthotopic xenograft implantation of GBM cells into immunodeficient mice. Patient-derived orthotopic glioblastoma xenograft models using surgical samples of GBM from patients reproduce the histopathology of human glioblastomas. The advantage of the orthotopic GBM xenograft model, compared to subcutaneous xenograft implantation model, is that implanting GBM cells into their anatomical origin equivalent within a host animal provides a biologically suitable site for glioma-brain interactions and maintains genomic characteristics of original human GBMs. Soeda et al. [23] established patient-derived several subclones from a single tumor of a patient as GBM xenograft model. Differences of cell morphology, invasiveness, progression, and proliferative activities, which represented the glioblastoma heterogeneity, were revealed in a mouse model featuring orthotopic xenografts of the subclones [23]. The subclones exhibiting more invasive and extensive infiltration induced higher mortality. The nestin-expressing cells are able to differentiate into multiple cell types in CNS development, acting like neuroepithelial stem cells. Thus, nestin is a marker of NSCs or neural progenitor cell [57, 58]. Soeda et al. [23] demonstrated that the nestin-expressing NSC-like human GBM cells were highly invasive and showed diffuse infiltration into the brain, including to contralateral hemispheres via the corpus callosum (Figure 5).

Recently, induced pluripotent stem (iPS) cells derived from human fibroblasts have been used to reproduce glial tumorigenesis. In vivo transplantation of transformed neural iPS cells produced highly invasive tumors containing undifferentiated stem cells, and this model has been used to screen the effectiveness of anticancer compounds and revealed specific molecules targeting transformed neural iPS cells [59].

## 6. Conclusion

Specific molecular parameters, in addition to traditional histopathological analysis, have been used to define tumor classification in the revised 4th edition of the WHO Classification of CNS tumors, published in 2016. Indeed, a large subset of glial tumors is now defined based on diagnostics of isocitrate dehydrogenase (IDH) mutation and 1p/19q codeletion, and histone H3 K27M mutation appears to be a fundamental event in diffuse infiltration of glioma cells in DIPG. As described in this review, currently GC is the only subtype of malignant glioblastoma developing a specific growth pattern. However, careful histomorphological examination is still important since, for example, the neoplastic cells within the GC can provide valuable insight into the mechanisms underlying glioma invasion. Furthermore, for example, morphological features such as “secondary structures of Scherer” are still important as diverse phenotypes of IDH(+) or IDH(-) glioma.

Detailed histopathological analysis based on the combination of molecular parameters with traditional analytical methods should be used for evaluating efficacy of targeted therapies against cellular and genetic heterogeneity within the invasive and resistant glioblastoma.
